# Sex‐Specific Differences in Cardiovascular Adaptations and Risks in Elite Athletes: Bridging the Gap in Sports Cardiology

**DOI:** 10.1002/clc.70006

**Published:** 2024-09-04

**Authors:** Siamak Afaghi, Fatemeh Sadat Rahimi, Pegah Soltani, Arda Kiani, Atefeh Abedini

**Affiliations:** ^1^ Chronic Respiratory Diseases Research Department National Research of Tuberculosis and Lung Disease Institution Tehran Iran

**Keywords:** arrhythmia, athletes, cardiac remodeling, cardiovascular adaptations, sex differences

## Abstract

**Background:**

The growing participation of women in competitive sports necessitates a comprehensive understanding of sex‐specific cardiovascular adaptations and risks. Historically, research has predominantly focused on male athletes, leaving a gap in knowledge about the unique cardiovascular dynamics of female peers.

**Hypothesis:**

we hypothesized that female athletes exhibit distinct cardiovascular adaptations and face different risks, influenced by physiological, hormonal, and structural differences.

**Methods:**

A systematic review of the literature was conducted, analyzing studies on cardiovascular responses and adaptations in athletes. Data were extracted on hemodynamic changes, autonomic and neural reflex regulation, cardiac remodeling, and arrhythmias. Comparative analyses were performed to identify sex‐specific patterns and discrepancies in cardiovascular health outcomes.

**Results:**

We revealed considerable sex differences in cardiovascular adaptations to athletic training. Female athletes generally have longer QT intervals, greater sinoatrial node automaticity, and enhanced atrioventricular node function compared to males. They also exhibit lower sympathetic activity, lower maximal stroke volumes, and a tendency toward eccentric cardiac remodeling. Conversely, male athletes are more prone to concentric hypertrophy and higher incidences of bradyarrhythmia and accessory pathway arrhythmias. Female athletes are more likely to experience symptomatic atrial fibrillation and face higher procedural complications during catheter ablation.

**Conclusions:**

Our findings underscore the necessity for sex‐specific approaches in sports cardiology. Recognizing and addressing these differences could enhance performance and reduce adverse cardiac events in athletes. Future research should focus on developing tailored screening, prevention, and treatment strategies to bridge the knowledge gap and promote cardiovascular health in both male and female athletes.

## Graphical abstract

The current study examines sex‐specific cardiovascular adaptations and risks among elite athletes, revealing distinct differences between male and female responses to training. Key findings include cardiac remodeling variations, arrhythmia risks, and exercise‐induced hemodynamic compliance, highlighting necessities for tailored sex‐specified approaches in sports cardiology to enhance performance and reduce adverse cardiac events.

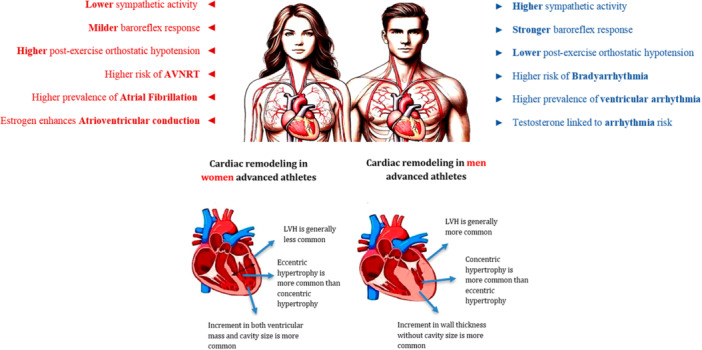

**Abbreviations**
AF: atrial fibrillation
AV: atrioventricular
AVO_2_
: arteriovenous oxygen difference
BP: blood pressure
CO: cardiac output
EDV: end‐diastolic volume
ESV: end‐systolic volume
ERP: early repolarization pattern
HCM: hypertrophic cardiomyopathy
HR: heart rate
JWI: J‐wave interval
LA: left atrium
LV: left ventricle
LVH: left ventricular hypertrophy
MVP: mitral valve prolapse
NICM: non‐ischemic cardiomyopathy
PVC: premature ventricular contraction
RV: right ventricle
SBP: systolic blood pressure
SCD: sudden cardiac death
SV: stroke volume
SVR: systemic vascular resistance
TWI: T‐wave inversion
VA: ventricular arrhythmias
VO_2_
: oxygen consumption
VO_2_max: maximal oxygen uptake

## 1 Introduction

In recent decades, there has been a consistent rise in the number of adults worldwide engaging in physical activity, observed across both developed and developing countries [1]. This trend is part of a larger global movement aimed at incorporating fitness into daily routines to mitigate the risk of metabolic‐related disorders and consequent cardiovascular disease, while also enhancing mental health, cognitive function, and overall quality of life [2]. Concurrently, the number of elite athletes has surged, catalyzing the development of a specialized field within cardiovascular medicine known as “sports cardiology” [3]. This discipline is dedicated to addressing the unique cardiovascular requirements and challenges faced by this population [3].

Notably, the participation of female athletes in competitive sports has seen a remarkable increase over the years. For instance, the proportion of women athletes in the Olympic Games has escalated from 23% in 1984 in Los Angeles to 49% in 2021 in Tokyo [3]. Despite this, the extant body of research investigating the cardiovascular impacts of exercise has predominantly concentrated on male athletes, with female athletes receiving comparatively limited attention.

Moreover, despite burgeoning interest in the biological underpinnings of sex differences in cardiovascular events, there remains a paucity of definitive evidence elucidating how physical activity may differentially influence cardiovascular health outcomes between genders [4]. Disparities in anthropometrics, physiological parameters, sex hormones, biochemical profiles, and psychological factors between genders contribute to a complex interplay of variables that affect cardiovascular adaptation to athletic training [4, 5].

Given these complexities, our review aims to provide an examination of sex‐specific characteristics in hemodynamic adaptation, autonomic and neurological reflexes, cardiac diagnostics, cardiac remodeling, and sport‐related cardiac pathologies. We also elucidate the mechanisms underlying these differences and identify critical gaps in the current knowledge that warrant further investigation. Addressing these gaps will foster a more equitable approach to cardiovascular health in athletes, ultimately enhancing performance and reducing the risk of adverse cardiac events.

## 2 Search Strategy

The search strategy of the current review involved a comprehensive search of electronic databases including PubMed, Scopus, Web of Science, and Embase. The search was limited to studies published between January 2000 and March 2024. A broad range of keywords and MeSH terms were used to capture relevant studies. These terms were categorized into two main groups: 1. Sex and Gender Differences Keywords, including “sex differences,” “gender differences,” “male vs female,” “sex‐specific,” “gender‐specific,” “biological sex,” “hormonal differences,” “gender disparities,” “female athlete,” “male athlete,” “sex‐based analysis,” “gender‐based analysis”; and 2. Cardiovascular Keywords, including “cardiovascular adaptations,” “cardiac remodeling,” “arrhythmia,” “hemodynamic response,” “left ventricular hypertrophy,” “sudden cardiac death,” “electrocardiogram variations,” “cardiac output,” “stroke volume,” “QT interval,” “sinoatrial node,” “atrioventricular node,” “cardiac electrophysiology,” “ventricular function,” “autonomic nervous system,” “sympathetic activity,” “baroreflex,” “myocardial strain,” “exercise‐induced cardiac changes.” Boolean operators (AND, OR, and NOT) were employed to combine search terms effectively and ensure the retrieval of a comprehensive set of relevant articles. Selection criteria studies were included if they met the following criteria: (1) original research articles or systematic reviews focusing on cardiovascular adaptations or risks in athletes; (2) studies that included both male and female athletes, allowing for direct comparison of sex‐specific cardiovascular responses; (3) studies that involved elite or competitive athletes, defined as individuals who participate in sports at a national or international level; (4) studies published in peer‐reviewed journals in English; and (5) studies providing quantitative data on cardiac function, structure, or incidence of cardiovascular events. Further, articles were excluded if they: (1) focused exclusively on non‐athlete populations; (2) included participants with known cardiovascular diseases or pre‐existing conditions that could confound the results; (3) were case reports, editorials, conference abstracts, or expert opinions without original data; (4) did not provide sex‐specific analysis or failed to report data separately for male and female athletes; (5) were published in languages other than English, where translation was not feasible; and (6) studies with insufficient methodological quality.

### 2.1 Study Selection Process

The initial search yielded a total of 645 articles. After removing duplicates, 452 articles were screened based on titles and abstracts. Two independent reviewers performed the screening, with discrepancies resolved by a third reviewer. A total of 167 articles were selected for full‐text review. Each article was evaluated for eligibility based on the predefined inclusion and exclusion criteria. Following the full‐text review, 50 articles were deemed suitable for inclusion in the current review.

## 3 Sex Differences in Hemodynamic Adaption

In human physiology, the increased energy demand and oxygen consumption (VO_2_) by muscles during exercise lead to a proportional increase in cardiac output (CO) [6]. Specifically, for every 1 L/min increase in VO_2_ during exercise, CO increases by approximately 1 L/min, reaching up to 6 L/min (average CO at rest is about 5 L/min) [7]. CO can be determined by two interrelated contexts: first, CO is expressed as heart rate (HR) multiplied by stroke volume (SV), where SV is the difference between end‐diastolic volume (EDV) and end‐systolic volume (ESV) [6]. EDV and ESV represent preload and afterload, respectively. Thus, CO can be re‐expressed as HR × (EDV − ESV). Favorably, increases in HR, SV, and CO during exercise prevent a decline in arterial blood flow due to systemic vasodilation, which occurs in response to metabolic‐induced vasodilators (e.g., lactate and carbon dioxide) [6, 7]. Blood pressure (BP) stabilization is modulated by neuromuscular and neurovascular pathways, including arterial baroreflex and the autonomic nervous system. Second, according to the Fick principle, CO equals total oxygen consumption divided by the arteriovenous oxygen difference (AVO_2_) [8]. AVO_2_ is the difference between oxygen content in arterial blood and mixed venous blood and depends on both the concentration and oxygen affinity of hemoglobin: AVO_2_ = hemoglobin × 1.34 × hemoglobin oxygen saturation [8]. Therefore, CO can be interpreted as total oxygen consumption divided by (hemoglobin × oxygen saturation) [8, 9]. Using these formulas, cardiovascular workload adjustment during physical activity relies on HR, preload, afterload, hemoglobin concentration, and oxygen saturation. Additionally, the rate of oxygen utilization by target muscles, termed “oxygen extraction fraction,” varies by exercise type (aerobic vs. endurance) and can further drive increases in CO [10].

Accumulating evidence indicates that women have lower sympathetic activity and peripheral artery resistance compared to men [11]. Accordingly, women exhibit lower resting systolic BP (SBP) levels and a milder response to baroreflex buffering compared to age‐matched men [3, 11]. This pattern is also observed among female athletes compared to their male counterparts [12]. Furthermore, during recovery from dynamic exercise, women demonstrate a lower capacity for vasoconstriction in the arteriolar bed compared to men, resulting in a greater risk of postexercise orthostatic hypotension due to a lower increase in systemic vascular resistance (SVR) [13]. This effect is particularly noticeable during inactive recovery, where female athletes exhibit a greater reduction in CO without adequate arteriolar constriction compared to men [8, 13]. However, these sex differences may not be extrapolated to elite athletes [14]. The lower sympathetic tone and vasodilatory tendency in women are even more pronounced during their reproductive lifespan compared to the postmenopausal state [15]. It is believed that sex hormones partially account for the lifelong sex differences in BP adaptation [13]. The arterial relaxant effect of estrogen is well established in prior studies [15]. However, sex disparities in BP response appear to be multifactorial rather than solely hormone dependent. For instance, most of the previous trials did not show promising results for hormone replacement therapy in attenuating arterial stiffness or prehypertension during the postmenopausal state [16]. Additionally, residual confounders, such as socioenvironmental factors and exercise type/duration, can affect sex differences in BP response to physical stress [17].

Another sex difference in hemodynamic adaptation is the lower maximal SV observed in women compared to men. The increase in SV during exercise occurs due to an increase in preload and a decrease in afterload, driven by increased cardiac inotropism [13]. The magnitude of changes in preload and afterload during exercise is reported to be similar between sexes [15]. Thus, the lower maximal SV in women could be attributed to a lower baseline volume of cardiac chambers rather than dynamic changes in ESV and EDV [7]. Specifically, women have a lower left ventricular (LV) capacity compared to men, which also explains the lower CO observed in women based on the CO = SV × HR formula [7]. Notably, HR does not exhibit sex‐related differences, as maximal HR during exercise is generally age‐dependent rather than sex‐dependent [14, 18]. Some studies suggest that SV, even when adjusted for body surface area, is lower in women compared to men, indicating that the lower overall SV in women may be independent of their smaller body size [19]. Differences in body composition (e.g., higher muscle mass in men compared to higher adiposity in women) may also affect maximal SV and CO during dynamic activity [19]. Furthermore, there appears to be a sex‐related difference in the SV increment slope during physical activity. Male athletes exhibit a gradual linear increase in SV without a significant plateau until maximal workload is reached, whereas female athletes, even at the elite level, reach a plateau in SV increment during submaximal workload [13, 20].

Recent research indicates that female athletes exhibit greater LV twist mechanics, potentially compensating for smaller ventricular volumes and aiding efficient CO during exercise. This compensation mechanism may partly explain why female athletes can achieve high‐performance levels despite smaller cardiac dimensions [21].

Another notable sex disparity is the lower AVO_2_ in women compared to men [19]. As previously mentioned, AVO_2_ depends on hemoglobin concentration, which is significantly lower in women [11]. Consequently, female athletes have a lower oxygen supply for the same blood flow compared to their male counterparts. Additionally, since CO, a major factor in oxygen transport, is lower in women, maximal oxygen uptake by muscles (VO_2_max) during exercise is lower in women, even after adjusting for age, fitness levels, and body surface area [13, 14]. Despite these differences, studies have shown that both sexes exhibit similar changes in the VO_2_max to CO ratio, suggesting a similar response to oxygen demand during exercise [3, 20]. With increasing age, the decline in HR and SV leads to a decrease in maximal CO and subsequent VO_2_max during exercise. This age‐related phenomenon does not appear to be significantly influenced by sex, although some evidence suggests that the age‐associated reduction in CO and VO_2_max occurs with greater intensity in men compared to women [22].

**Figure 1:**
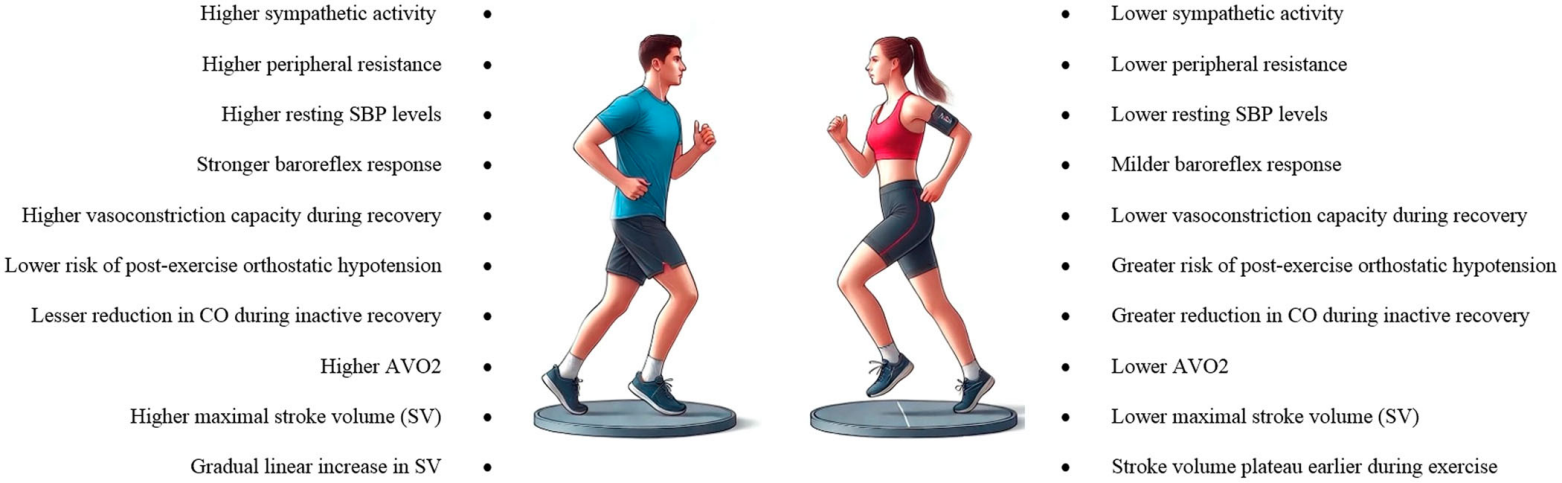
The sex differences in physiological response to physical activity.

Additionally, recent studies have highlighted that hormonal fluctuations throughout the menstrual cycle can influence cardiovascular responses during exercise in women, with variations in VO_2_max, HR, and CO observed across different phases of the cycle [21]. This underscores the importance of considering menstrual cycle phases in research and training programs for female athletes to optimize performance and cardiovascular health. Figure 1 illustrates the overview of the mentioned findings.

## 4 Sex Differences in Neural Reflex Regulation

Three neural adaptations regulate the hemodynamic response to physical activity: the “central command reflex,” responsible for activating the sympathetic nervous system and recruiting motor units; the “pressor reflex,” which adjusts mean arterial pressure (MAP) and HR based on peripheral mechanical and metabolic triggers from working muscles; and the “arterial baroreflex,” which maintains the balance between the sympathetic and parasympathetic nervous systems modulated by prior reflexes [23, 24].

Several studies have explored potential sex differences in these regulatory mechanisms. Recent research indicates distinct patterns of activation in the central command reflex within the insular cortex between sexes [13]. Specifically, women exhibit more right‐sided insular activation, while men show more left‐sided activation during exercise [23]. This lateralization may have implications for how each sex processes and responds to physical exertion [18, 25].

Studies also suggest that both metabolic‐reflex and mechano‐reflex components of the exercise pressor reflex are reduced in women compared to men, resulting in a lower increase in BP and sympathetic nerve activity during exercise. Recent findings indicate that postmenopausal women exhibit an augmented exercise pressor reflex, which can impair muscle blood flow due to excessive sympathetic vasoconstriction. Estrogen replacement therapy has been shown to attenuate these effects, highlighting the role of sex hormones in modulating this reflex [26].

Research on the arterial baroreflex has produced mixed results, with some studies reporting reduced cardiac baroreflex sensitivity at rest in women, while others have found no significant sex differences [18]. Current studies emphasize the need to consider the interaction between aging and sex hormones in understanding these differences. For instance, testosterone levels in men have been identified as predictors of the exercise pressor response, suggesting that both male and female hormones significantly influence neural cardiovascular control during exercise [26].

Overall, understanding these sex‐specific differences in neural reflex regulation is crucial for optimizing cardiovascular health and performance in both male and female athletes, especially given the potential implications for personalized training and therapeutic strategies. Further investigations are warranted to provide a more comprehensive understanding of these mechanisms and to inform targeted interventions.

## 5 Sex Differences in Cardiac Remodeling

**Figure 2:**
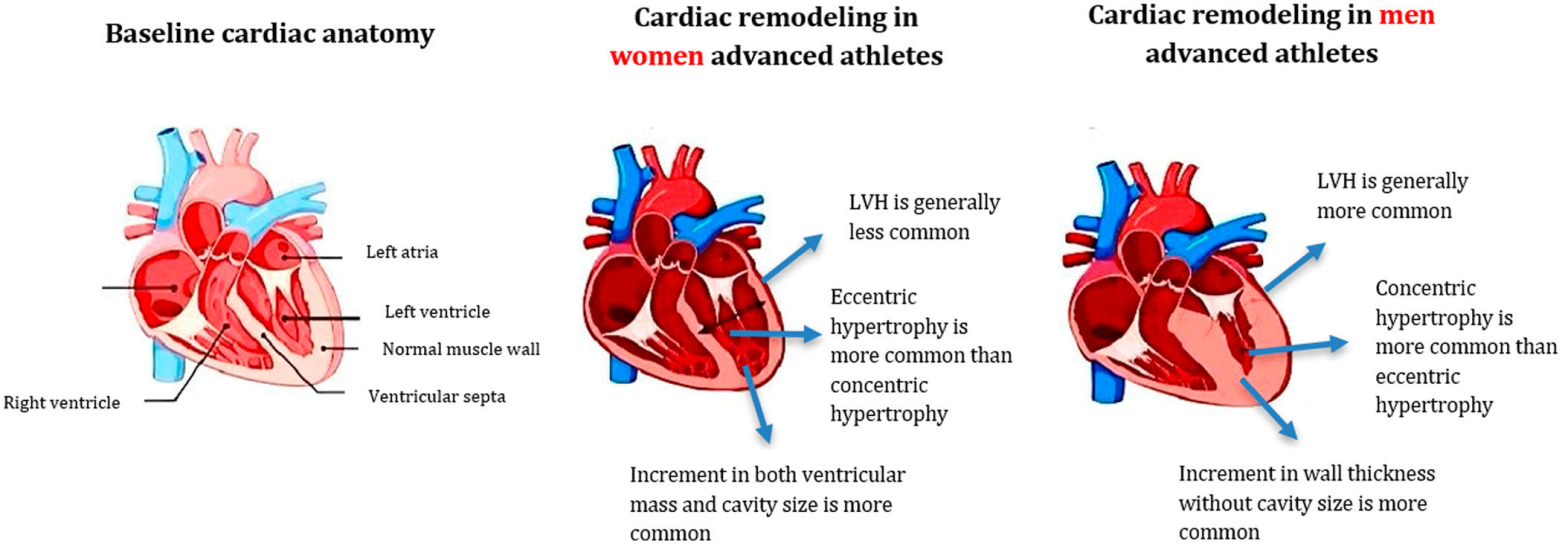
The sex differences in cardiac remodeling as a response to physical activity among advanced athletes.

Cardiac adaptation to regular exercise is influenced by a variety of factors including the type and intensity of exercise, age, ethnicity, and sex. Studies have indicated that most athletes exhibit normal LV geometry, with a higher prevalence among female athletes compared to their male counterparts (Figure 2). Recent research underscores the importance of differentiating between physiological and pathological changes in cardiac structure for both sexes [27, 28].

Exercise‐induced cardiac remodeling can result in changes to cavity size and wall thickness, manifesting as either eccentric or concentric hypertrophy. Eccentric hypertrophy involves an increase in both LV mass and cavity size due to volume overload and elevated diastolic wall stress, whereas concentric hypertrophy is characterized by increased wall thickness without significant changes in cavity size [29, 30]. In female athletes, eccentric hypertrophy is more common in response to dynamic exercises, while male athletes are more likely to develop concentric hypertrophy [13, 30]. LV wall thickness greater than 12 mm and LV cavity enlargement are rare among female athletes. Additionally, left atrial (LA) enlargement, common among elite athletes, is less frequently observed in female athletes compared to males. Concentric changes in female athletes may suggest pathological cardiomyopathies rather than physiological adaptations [30].

The pattern of LV remodeling in male athletes can be partially attributed to higher testosterone levels [23]. Male athletes also tend to experience higher peak BP during exercise, which contributes to LV hypertrophy, despite similar peak HRs between sexes. Female athletes, on the other hand, generally exhibit lower BP, CO, and absolute peak workload [4, 21].

Right ventricular (RV) remodeling, like LV changes, also occurs in response to regular exercise. Echocardiographic studies have shown that both RV size and wall thickness increase in athletes of both sexes, though the extent of these changes is typically less in female athletes. However, the lack of standardized indices for RV remodeling evaluation in athletes necessitates further research to confirm these findings [31].

Overall, cardiac remodeling is more pronounced in male athletes, particularly in the LV. When adjusting for body surface area, both LV and RV sizes are lower in male athletes compared to female athletes, highlighting the importance of considering body composition in these assessments. Factors such as differences in training patterns, body surface area, height, lean body mass, and allometric scaling significantly influence right and left heart remodeling [14, 31].

## 6 Sex Differences in Electrocardiogram

Regular exercise induces normal variations in the 12‐lead ECG of athletes. If accompanied by other pathological symptoms or ECG patterns, these variations require further evaluation [32]. These normal variations result from both structural adaptations of the heart—discussed earlier as cardiac remodeling—and the nerve conduction system of the heart, prominently the vagus nerve, in response to regular physical activity [23]. A list of normal and pathological ECG patterns has been described in previous studies, but the differences between male and female athletes are still under investigation [33].

Early repolarization pattern (ERP), defined as J‐point elevation of 0.1 mV or more in lateral and/or inferior leads, is a common finding in the ECGs of healthy young individuals and has been positively correlated with physical activity and male sex. Recent studies have confirmed that the prevalence of ERP is higher in male athletes compared to female athletes [34]. Individuals with ERP in their ECGs are at increased risk of sudden cardiac death (SCD) and ventricular and atrial arrhythmias [35].

Cardiac arrhythmias such as ventricular arrhythmias (VAs) (couplets, triplets, and non‐sustained ventricular tachycardia), atrial tachyarrhythmias (supraventricular tachycardia, atrial fibrillation [AF], atrial flutter), and frequent premature ventricular contractions (PVCs) are common abnormal findings in athletes' ECGs [36]. However, the difference between sexes in these arrhythmias is not well documented. Vagotonia, more prevalent in physically active individuals than in the general population, contributes to the higher prevalence of J‐point elevation in athletes [37]. Endocrine and transmembrane voltage gradient differences between sexes have been suggested as reasons for the higher prevalence of ERP, but data on these mechanisms in physically active individuals are lacking [29, 36].

Another common finding in the ECGs of male athletes compared to female athletes is widened or deep Q waves [38]. Abnormal Q waves in the lateral and inferior leads are often not considered pathological variations but are attributed to physiological LVH and technical factors such as lead misplacement. However, they can sometimes indicate structural heart diseases such as hypertrophic cardiomyopathy (HCM) [11, 36, 38]. Studies have shown that female athletes exhibit a lower increase in LV wall thickness and rarely exceed the maximum wall thickness defined for physiological LVH [14]. The upper limit of LV wall thickness in athletes, known as physiological LVH, is reported to be 16 mm. While a thickness of 13 mm or higher, suggestive of HCM, is rare among male athletes compared to non‐athletes, recent studies report that a wall thickness of 12 mm or higher is even less common among female athletes, which could explain the pathological Q waves more commonly seen in male athletes [38, 39].

Evidence of RV and LV hypertrophy is more frequently found in the ECGs of male athletes [21, 29]. Anterior T wave inversion (TWI) is more common in female athletes, while deep or lateral TWI and a combination of anterior TWI and J‐point elevation of more than 0.1 mV are more prevalent in male athletes [37, 38]. Female athletes also tend to have shorter PR intervals and QRS durations compared to male athletes [37].

These findings underscore the necessity for sex‐specific criteria when interpreting ECGs in athletes to accurately differentiate between physiological adaptations and potential pathologies.

## 7 Sex Differences in Cardiac Arrhythmias

Sex differences in electrophysiological properties are well documented. Table 1 compares an overview of male and female athletes regarding arrhythmia risks, types, and treatment outcomes. Women typically exhibit greater sinoatrial node automaticity, enhanced atrioventricular node function, longer infra‐Hisian conduction times, and prolonged ventricular action potential durations [31, 37, 38]. Conversely, studies indicate that male athletes have a higher susceptibility to bradyarrhythmia and accessory pathway arrhythmias, such as high‐degree atrioventricular block, while female athletes are more prone to atrioventricular nodal re‐entry tachycardias (AVNRT) [40].

**Table 1:** Comparison of male and female athletes regarding arrhythmia risks, types, and treatment outcomes.

Arrhythmia type and complications	Men	Women
General electrophysiological properties	Shorter QT interval postpuberty, faster repolarization, increased risk of ventricular arrhythmias in older age due to higher QT dispersion	Longer QT interval, greater sinoatrial node automaticity, enhanced AV node function, prolonged ventricular action potential durations
Atrial fibrillation (AF)	Higher incidence in general; vigorous endurance exercise increases risk; often asymptomatic	Higher prevalence in older age due to longer life expectancy; mitral valve prolapse increases risk; more symptomatic with palpitations, shortness of breath, fatigue; higher recurrence and complications post‐catheter ablation
AV nodal re‐entry tachycardias (AVNRT)	Less common	More common in female athletes
Idiopathic ventricular arrhythmias (VA)	Less common, more associated with structural heart disease	More common due to myocardial tissue properties and sex hormone influence
Risk factors (alcohol, hypertension, etc.)	A similar association with AF development as women	A similar association with AF development in men
Exercise‐related risk	Higher risk of AF with vigorous endurance exercise; prone to ventricular arrhythmias during intense exercise	Mitral valve prolapses linked to new‐onset AF; higher symptomatic AF; benefit from targeted interventions for AF and AVNRT
Hormonal influence	Testosterone accelerates ventricular repolarization, associated with adverse cardiac remodeling and increased arrhythmia risk	Estrogen reduces maladaptive cardiac remodeling and fibrosis, decreasing VA incidence in premenopausal women; progesterone is protective against severe ventricular arrhythmias and sudden cardiac death during pregnancy
Sudden cardiac death (SCD)	Higher incidence compared to women, especially in older age	Lower incidence overall, but risk increases postpartum due to a drop in progesterone levels
Supraventricular tachycardias (SVT)	Less common	More likely to experience SVT such as AVNRT, increased risk with structural changes in the heart during pregnancy and menopause
Long QT syndrome (LQTS)	Shorter QT interval due to higher testosterone levels; risk of arrhythmias increases with age	Longer QT interval; risk of cardiac events varies with menstrual cycle, lower during pregnancy but increases postpartum
Torsades de Pointes	Less common	More susceptible due to longer QT interval, especially in those with LQTS or during hormonal fluctuations
Implantable cardioverter‐defibrillator (ICD) outcomes	More likely to receive ICDs, better outcomes with fewer complications	Less likely to receive ICDs; higher complication rates postimplantation
Treatment of atrial fibrillation	Equally likely to receive antiarrhythmic drugs; more likely to undergo catheter ablation with better outcomes	Less likely to receive invasive treatments; higher risk of complications like cardiac tamponade; primary AV‐nodal ablation more common due to comorbidities and older age at diagnosis
Ventricular tachycardia (VT)	VT associated with structural heart disease more common; higher comorbidity burden	Idiopathic VT is more common, especially RVOT‐VT; worse VT–free survival despite favorable baseline characteristics
Management and outcomes	Better outcomes with fewer complications for catheter ablation; more often receive cardiac resynchronization devices	Higher recurrence rates post‐catheter ablation; more procedural complications; less likely to be referred for invasive procedures

In terms of AF, a common arrhythmia with significant morbidity and mortality, there is substantial evidence of sex‐based differences in its mechanisms, treatment responses, and outcomes within the general population [41, 42]. However, it remains unclear whether these sex‐specific differences extend to the relationship between AF and long‐term exercise, as most studies have primarily focused on male athletes. Moderate regular exercise seems to mitigate AF risk in both sexes, while vigorous endurance exercise may increase it, especially in male athletes [43, 44]. Some research suggests a J‐shaped relationship between the risk of AF and the intensity of endurance exercise [45, 46].

In athletes who develop AF without significant cumulative exercise exposure, mitral valve prolapse (MVP) has been linked to new‐onset AF, with female athletes being at higher risk than males [47]. Classic risk factors for AF, such as alcohol consumption, hypertension, thyrotoxicosis, and ischemic heart disease, do not exhibit sex differences in their association with AF development [48]. Female athletes are more likely to experience symptomatic AF, presenting with palpitations, shortness of breath, and fatigue, whereas asymptomatic AF is more commonly reported in male athletes [45, 49].

Sex differences also extend to the management and outcomes of cardiac arrhythmias [45]. Women athletes undergoing catheter ablation for AF are typically older than men and show higher rates of AF recurrence and procedural complications, such as cardiac tamponade [9, 50]. This finding may be at least in part due to the higher prevalence of non‐pulmonary vein–mediated AF in women, which is more challenging to treat with standard pulmonary vein isolation techniques [51]. Additionally, VAs display significant sex differences [52]. For instance, idiopathic VAs are more common in women, whereas men are more likely to experience VA associated with structural heart disease. These variations are partly attributed to differences in myocardial tissue properties and the influence of sex hormones [37, 52].

Furthermore, sex hormones play a critical role in modulating arrhythmia risk. Estrogen provides cardioprotective effects by reducing maladaptive cardiac remodeling and fibrosis, thereby decreasing VA incidence in pre‐menopausal women, while testosterone has been associated with adverse cardiac remodeling and increased arrhythmia risk in men [13, 37].

Some studies underscore the sex‐specific differences in athletes' cardiac arrhythmias for developing tailored screening, prevention, and treatment strategies for athletes. For instance, male athletes are more prone to ventricular arrhythmias during intense exercise, which necessitates regular monitoring and possibly more stringent cardiovascular screening protocols [37, 41]. Conversely, female athletes might benefit more from targeted interventions aimed at managing AF and AVNRT, emphasizing the need for personalized medical approaches [21, 41].

## 8 Sex Differences in SCD

Extensive epidemiological research indicates that women have a lower incidence of SCD during physical activity, irrespective of age and athletic proficiency [4, 31, 37]. Younger female athletes exhibit fewer cardiomyopathies linked to SCD compared to their male counterparts [4]. Additionally, females who experience SCD typically have structurally normal hearts [4, 31, 37]. Women with structural heart diseases are also generally at a lower risk of SCD than men, though conditions like MVP may significantly heighten this risk in females [33, 34, 48].

Studies on elite athletes showed that female athletes tend to have smaller LV wall thickness and larger LV chambers, indicating that eccentric hypertrophy is more common in women, whereas concentric hypertrophy predominates in men [27, 29]. This suggests that the type of cardiac remodeling seen in female athletes might offer protection against SCD. Hormonal differences, such as the contrasting effects of estrogen and testosterone on myocardial hypertrophy and adrenaline levels during intense exercise, likely contribute to this protective mechanism [18]. Moreover, the cardioprotective role of estrogen, which reduces maladaptive cardiac remodeling and fibrosis and stabilizes electrophysiological properties, further underscores the importance of hormonal influences in sports cardiology [37].

Recent research has also highlighted that women have more active enzymatic reactions related to energy substrates, which can prevent myocardial fibrosis [53]. This points to a complex interplay of biomechanical factors, including epigenetics, hormones, and biochemical pathways, contributing to the reduced risk of sport‐related SCD in women.

The implications of these sex‐specific differences are crucial for the field of sports cardiology. Understanding these protective mechanisms in women can guide the development of tailored screening and intervention strategies. For example, male athletes may benefit from more rigorous cardiovascular monitoring and screenings for conditions such as HCM and arrhythmogenic RV cardiomyopathy (ARVC), which are more frequently associated with SCD in men due to their tendency for structural heart abnormalities and arrhythmias under high physical stress. Conversely, female athletes might need focused screenings for conditions like MVP and AF. Given these conditions' contribution to increased SCD risk in women, particularly under intense physical activity, early identification and intervention are essential.

## 9 Conclusion

The increasing participation of women in elite sports has highlighted the critical need for a deeper understanding of sex‐specific cardiovascular adaptations and associated risks. Cumulating evidence underscores the importance of recognizing sex as a fundamental variable in sports cardiology, influencing not only athletic performance but also the risk of adverse cardiovascular events. The findings of this review emphasize the necessity for sex‐specific approaches in the evaluation, management, and treatment of athletes. It is clear that the one‐size‐fits‐all model in sports cardiology does not adequately address the unique physiological and hormonal differences between men and women. This gap in knowledge must be bridged to ensure optimal cardiovascular health and performance for all athletes, regardless of sex. To advance the field of sports cardiology and promote highly personalized medicine, we urge the scientific community to prioritize research that investigates sex‐specific cardiovascular dynamics in athletes. Future studies could aim to develop tailored screening protocols, prevention strategies, and plausible therapeutic interventions that account for the distinct cardiovascular profiles of male and female athletes. Enhanced research in this domain will not only contribute to safer athletic participation but also pave the way for innovations in personalized medicine, ultimately improving outcomes for all athletes.

## Conflicts of Interest

The authors declare no conflicts of interest.

## Ethics Statement

The authors have nothing to report.

## Data Availability

The data that support the findings of this study are available from the corresponding author upon reasonable request.
